# Inhibition of Adhesion Molecule Gene Expression and Cell Adhesion by the Metabolic Regulator PGC-1α

**DOI:** 10.1371/journal.pone.0165598

**Published:** 2016-12-16

**Authors:** Neri Minsky, Robert G. Roeder

**Affiliations:** Laboratory of Biochemistry and Molecular Biology, The Rockefeller University, New York, New York, United States of America; Institut de Genomique Fonctionnelle de Lyon, FRANCE

## Abstract

Cell adhesion plays an important role in determining cell shape and function in a variety of physiological and pathophysiological conditions. While links between metabolism and cell adhesion were previously suggested, the exact context and molecular details of such a cross-talk remain incompletely understood. Here we show that PGC-1α, a pivotal transcriptional co-activator of metabolic gene expression, acts to inhibit expression of cell adhesion genes. Using cell lines, primary cells and mice, we show that both endogenous and exogenous PGC-1α down-regulate expression of a variety of cell adhesion molecules. Furthermore, results obtained using mRNA stability measurements as well as intronic RNA expression are consistent with a transcriptional effect of PGC-1α on cell adhesion gene expression. Interestingly, the L2/L3 motifs of PGC-1α, necessary for nuclear hormone receptor activation, are only partly required for inhibition of several cell adhesion genes by PGC-1α. Finally, PGC-1α is able to modulate adhesion of primary fibroblasts and hepatic stellate cells to extracellular matrix proteins. Our results delineate a cross talk between a central pathway controlling metabolic regulation and cell adhesion, and identify PGC-1α as a molecular link between these two major cellular networks.

## Introduction

PPARγ co-activator 1α (PGC-1α) is a pivotal co-activator protein that associates with numerous transcription factors and increases their ability to induce expression of their cognate target genes [1, 2]. Deregulation of PGC-1α mRNA levels has been noted in obesity and several other disease states [1, 2]. A key attribute of PGC-1α is its ability to boost oxidative metabolism and enhance mitochondrial biogenesis [3]. PGC-1α can also induce tissue-specific programs such as hepatic gluconeogenesis [4], thermogenesis in brown adipose tissue (BAT) [5], and fiber-type switching in skeletal muscle [6]. PGC-1α is induced by a variety of physiological stimuli in the tissues where it acts, including exercise in muscle, cold in BAT, and fasting or diabetes in the liver [1, 2]. Mechanistically, PGC-1α induces gene expression via a strong transcriptional activation domain at its N terminus. This domain interacts with several lysine acetyltransferase complexes that include p300, 3'-5'-cyclic adenosine monophosphate (cAMP) response element-binding protein (CREB)-binding protein, and steroid receptor coactivator-1 [7]. Additionally, the C-terminal domain of PGC-1α interacts with the switch/sucrose nonfermentable (SWI/SNF) chromatin-remodeling complex through its interaction with BAF60a [8]. The C-terminal region of PGC-1α also interacts with the MED1/TRAP220 subunit of the Mediator complex, potentially facilitating Mediator recruitment and interaction with the transcription initiation machinery [9]. The ability of PGC-1α to co-activate nuclear hormone receptors depends on two N-terminal LXXLL motifs designated L2 and L3, involved in the interaction between PGC-1α and these transcription factors [10, 11].

While PGC-1α is a well described activator of metabolic pathways, previous studies carried out mainly in mouse muscle and myocytes suggested that PGC-1α may inhibit chronic inflammation. However, the mechanisms underlying these effects are poorly understood. Studies employing mice lacking PGC-1α specifically in muscle demonstrated the transcriptional induction of a few markers indicative of local or systemic inflammation [12, 13]. These inflammatory markers, such as IL-6 and TNFα, were elevated in skeletal muscle of muscle-specific PGC-1α knockout (KO) animals [12, 13]. Primary myotubes with a deletion of PGC-1α were reported to have higher levels of TNFα and IL-6 mRNAs than wild type. In addition, ectopic expression of PGC-1α in C2C12 cultured myotubes inhibited the expression of IL-6 and TNFα mRNAs [12]. These observations partly differ from other studies indicating that PGC-1α enhances, rather than reduces, basal TNFα and IL-6 expression in skeletal muscle [14]. Furthermore, mice with a muscle-specific PGC-1α knock-out had reduced plasma TNFα levels and skeletal muscle TNFα mRNA levels in response to LPS treatment [14].

While the molecular mechanisms that underlie these PGC-1α effects on inflammatory gene expression are incompletely understood, they have been previously postulated to involve regulation of reactive oxygen species by PGC-1α [15]. More recently, ectopic expression of PGC-1α has been demonstrated to repress the transcriptional activity of NFkB in cultured myotubes, thereby affecting NFkB-dependent transcription [16] and contributing, at least in part, to the anti-inflammatory activity of PGC-1α. Interestingly, our recent work revealed a direct inhibitory effect of PGC-1α on the major mammalian regulator of the heat shock response, Heat-shock factor 1 (HSF1), resulting in suppression of transcriptional programs related to heat shock protein expression, as well as others [17].

Here we show that PGC-1α can act to inhibit the expression of several cell adhesion molecules, including integrins and cadherins. By analyzing microarray data representing a variety of cell types we demonstrate the ability of PGC-1α to down-regulate a plethora of cell adhesion related genes; and we validate these observations at the mRNA and protein levels. Furthermore, we investigate the possible mechanism of the effects of PGC-1α on cell adhesion gene expression, and suggest that the effect is transcriptional and only partly involves the well-described ability of PGC-1α to associate with nuclear hormone receptors. Importantly, we also establish an effect of PGC-1α on cell adhesion to extracellular matrix protein-coated surfaces. Thus, by examining the effects of both endogenous and ectopically expressed PGC-1α, we highlight a yet unexplored facet of the activity of PGC-1α, a major metabolic regulator and transcriptional co-activator, and link it to cell adhesion.

## Results

**Fig 1:**
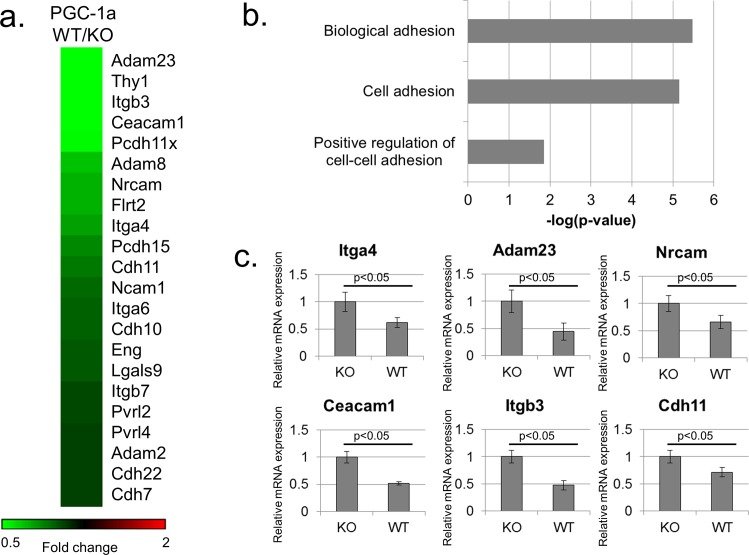
Endogenous PGC-1α inhibits cell adhesion gene expression in MEFs. (A) Microarray analysis of genes differentially expressed between PGC-1α KO and WT MEFs. Shown are cell adhesion related genes down-regulated 1.3 fold or more dependent on PGC-1α. (B) Gene annotation enrichment analysis of genes down-regulated 1.5 fold or more dependent on PGC-1α in MEFs. (C) RT-PCR validations of several cell adhesion genes identified in the microarray analysis as down-regulated dependent on PGC-1α. Readings were normalized to β-Actin mRNA.

As part of our efforts to explore the transcriptional landscapes regulated by PGC-1α, we looked into the possible effect of PGC-1α on mRNA levels in primary mouse embryonic fibroblasts (MEFs) derived from either wildtype (WT) or PGC-1α KO mice (Fig 1). With a cutoff of 1.5 fold change, 663 genes were up-regulated and 668 genes were down-regulated dependently on PGC-1α in these cells. Strikingly, numerous genes involved in cell adhesion and its regulation are down-regulated by PGC-1α in these cells (Fig 1A). These genes include integrins, cadherins, and proto-cadherins among others. Gene annotation enrichment analysis revealed a significant enrichment for terms related to cell adhesion among genes down-regulated by PGC-1α in MEFs (Fig 1B). Conversely, no significant enrichment was found for terms related to cell adhesion among genes up-regulated by PGC-1α in MEFs. In a validation of these observations, real-time PCR analysis on cDNA derived from these cells confirmed the PGC-1α-dependent down-regulation of several genes (Fig 1C). As the employed PGC-1α KO MEFs were derived from whole body PGC-1α KO mice, we wondered whether PGC-1α may play a cell-autonomous role in regulating cell adhesion genes. We further speculated that the effect may be manifested in human cells as well.

**Fig 2:**
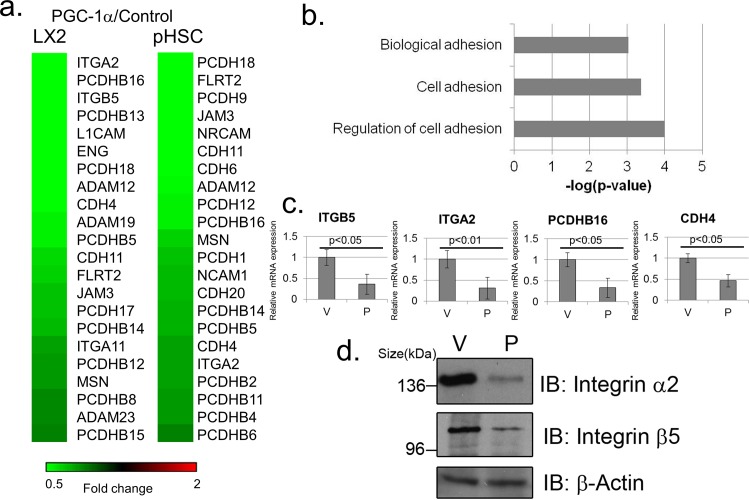
Ectopic expression of PGC-1α in human hepatic stellate cells down-regulates cell adhesion gene expression. (A) Microarray analysis of genes differentially expressed between PGC-1α overexpressing and control LX2 cells (left panel) or human primary hepatic stellate cells (right panel). Shown are cell adhesion related genes down-regulated 1.3 fold or more dependent on PGC-1α. (B) Gene annotation enrichment analysis of genes down-regulated 1.5 fold or more dependent on PGC-1α in LX2 cells. (C) RT-PCR validations of several cell adhesion genes identified in the LX2 microarray analysis as down-regulated dependent on PGC-1α. Cells were infected with either an empty vector control adenovirus (“V”) or a PGC-1α encoding adenovirus (“P”). Readings were normalized to β-Actin mRNA. (D) Western blot analysis of the indicated integrin proteins in LX2 cells. Cells were infected with either an empty vector control adenovirus (“V”) or a PGC-1α encoding adenovirus (“P”).

To address these points, we utilized an adenovirus vector to express PGC-1α in the human hepatic stellate cell line LX2 [18]. Reassuringly, in these cells as well, PGC-1α expression elicited an inhibitory effect on cell adhesion gene expression (Fig 2A, left panel). To further support a role for PGC-1α in down-regulating cell adhesion gene expression in human hepatic stellate cells, we utilized primary human hepatic stellate cells. A similar analysis in primary human hepatic stellate cells uncovered a similar pattern of cell adhesion gene expression inhibition upon PGC-1α expression (Fig 2A right panel). A gene annotation enrichment analysis based on the LX2 cell microarray analysis revealed a significant enrichment for terms related to cell adhesion among genes down-regulated by PGC-1α (Fig 2B). Real-time PCR analyses confirmed the microarray observations in LX2 cells (Fig 2C), as well as in primary human hepatic stellate cells (S1 Fig). In addition, we observed a PGC-1α dependent down-regulation of integrin α2 (encoded by the ITGA2 gene) and integrin 5 (encoded by the ITGB5 gene) expression at the protein level (Fig 2D). As fibroblasts, such as MEFs and hepatic stellate cells, are relatively unexplored systems with regard to PGC-1α activity, we looked at additional systems to examine the effects of PGC-1α on cell adhesion gene expression.

**Fig 3:**
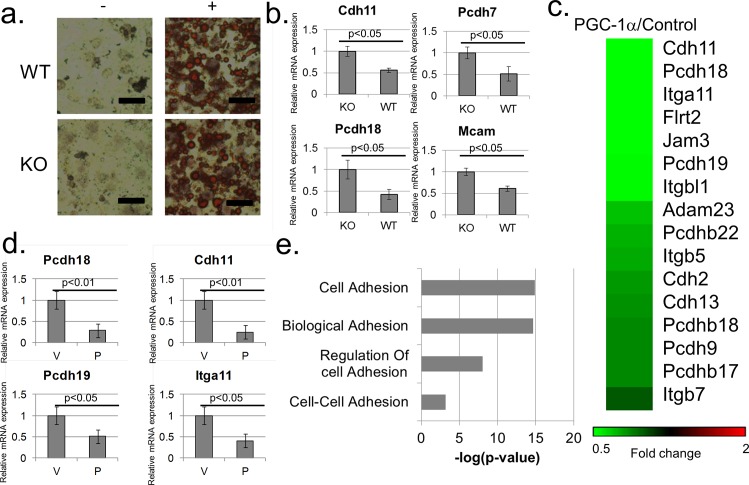
Regulation of cell adhesion gene expression by PGC-1α in brown adipocytes. (A) PGC-1α status does not affect brown adipocyte differentiation *in vitro*. KO or WT PGC-1α brown pre-adipocytes were either kept undifferentiated (“-“) or were differentiated (“+”) and stained with Oil-Red-O. Scale Bars, 50μm. (B) RT-PCR analysis of several cell adhesion genes down-regulated dependent on PGC-1α. Readings were normalized to β-Actin mRNA. (C) Microarray analysis of genes differentially expressed between PGC-1α overexpressing and control KO brown adipocytes. Shown are cell adhesion related genes down-regulated 1.3 fold or more dependent on PGC-1α. (D) Gene annotation enrichment analysis of genes down-regulated 1.5 fold or more dependent on PGC-1α in PGC-1α KO brown adipocytes infected with an adenovirus encoding PGC-1α. (E) RT-PCR validations of several cell adhesion genes identified in the infected KO PGC-1α brown adipocyte microarray analysis as down-regulated dependent on PGC-1α. Cells were infected with either an empty vector control adenovirus (“V”) or a PGC-1α encoding adenovirus (“P”). Readings were normalized to β-Actin mRNA.

Since PGC-1α has been traditionally regarded as a critical regulator of gene expression in brown adipocytes, we took advantage of previously established [19] immortalized brown preadipocytes derived from either WT or PGC-1α KO mice. First, as evident from Oil-Red-O staining and consistent with previous results, PGC-1α status in these cells did not affect differentiation of preadipocytes into mature adipocytes (Fig 3A). Importantly, as demonstrated by RT-PCR analyses in WT versus KO cells, the expression of several cell adhesion-related genes was inhibited by endogenous PGC-1α (Fig 3B). As these cells were previously subjected to microarray analysis [19], we examined the status cell adhesion genes in the publicly available data (GSE5041) from that analysis. Strikingly, many cell adhesion genes were found to be down-regulated by endogenous PGC-1α when comparing two WT and two KO PGC-1α differentiated brown adipocyte samples (S2A Fig). In addition, a gene annotation enrichment analysis revealed a significant enrichment for terms related to cell adhesion among genes down-regulated dependent on PGC-1α in this system (S2B Fig). To further investigate the impact of PGC-1α on cell adhesion gene expression in these cells, PGC-1α KO differentiated brown adipocytes were infected with an adenovirus encoding PGC-1α. A subsequent microarray analysis revealed that with a cutoff of 1.5 fold change, 929 genes were up-regulated and 843 genes were down-regulated dependently on PGC-1α in these cells. Importantly, the analysis uncovered many cell adhesion mRNAs that were inhibited by PGC-1α expression (Fig 3C), and these were validated by RT-PCR (Fig 3D). In addition, gene annotation enrichment analysis indicated a significant enrichment for terms related to cell adhesion among genes down-regulated by PGC-1α in these cells (Fig 3E). Notably, such enrichment was not detected among genes up-regulated by PGC-1α in these cells; conversely, a significant enrichment for terms related to mitochondrial respiration was detected, as expected, among genes up-regulated by PGC-1α (S3 Fig).

**Fig 4:**
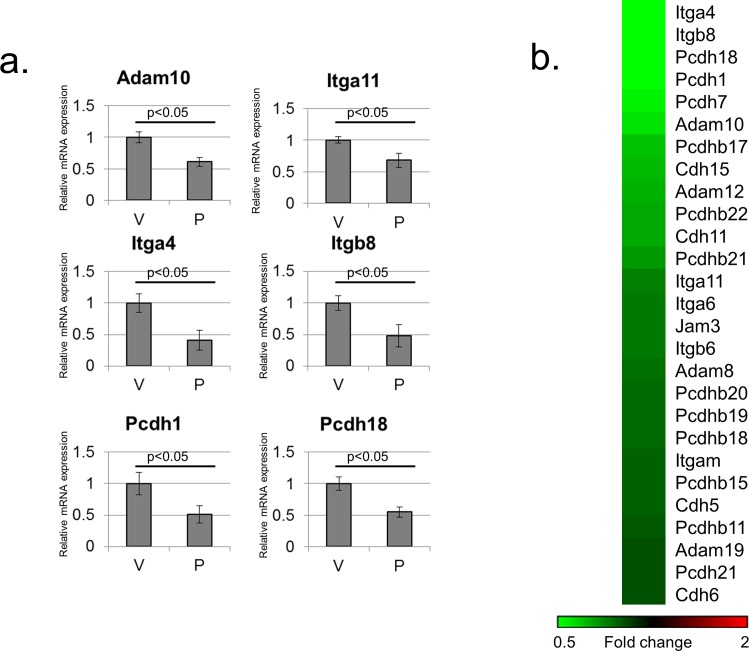
PGC-1α regulates cell adhesion genes in C2C12 myotubes. (A) RT-PCR analysis of several cell adhesion genes down-regulated dependent on PGC-1α in C2C12 cells. Cells were infected with either an empty vector control adenovirus (“V”) or a PGC-1α encoding adenovirus (“P”). Readings were normalized to β-Actin mRNA. (B) Reanalysis of publicly available microarray data from C2C12 cells (GSE51498). Shown are cell adhesion related genes down-regulated 1.3 fold or more dependent on PGC-1α.

An additional system widely used to study PGC-1α is muscle [20]. To address the possible effects of PGC-1α on cell adhesion gene expression in muscle cells, C2C12 cells were differentiated into myotubes in culture. Differentiated myotubes were infected with a control adenovirus or an adenovirus encoding PGC-1α, and mRNA was analyzed by RT-PCR. Notably, in this system as well, many cell adhesion gene mRNAs were down-regulated dependent on PGC-1α expression (Fig 4A). These results were further supported by a reanalysis of our previously published C2C12 microarray data (GSE51498), which revealed widespread inhibition of expression of cell adhesion-related genes (Fig 4B).

**Fig 5:**
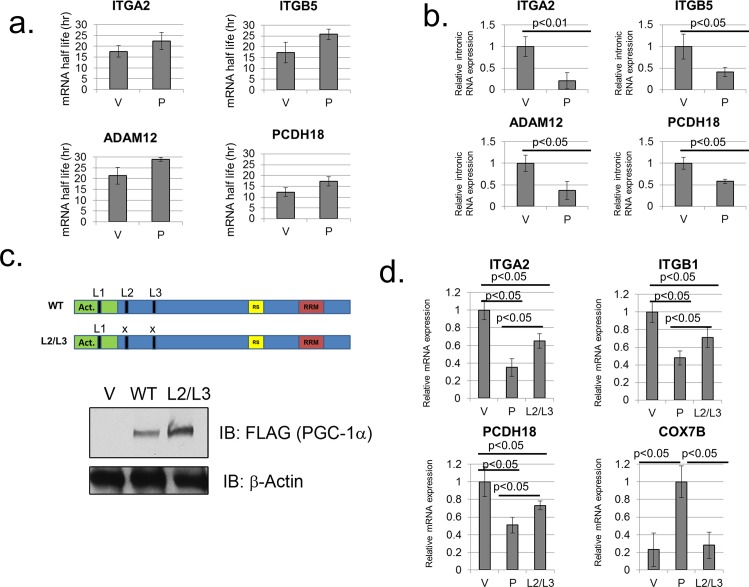
The LXXL motifs of PGC-1α are partly required for inhibition of cell adhesion gene expression, which is not mediated via an effect on mRNA stability. (A) PGC-1α does not destabilize cell adhesion gene mRNAs. LX2 cells were infected with either an empty vector control adenovirus (“V”) or a PGC-1α control adenovirus (“P”) and treated with 2μM Actinomycin D for 17hr or left untreated. RNA was extracted and subjected to RT-PCR analysis, and mRNA half-lives were calculated. (B) LX2 cells were infected with either an empty vector control adenovirus (“V”) or a PGC-1α control adenovirus (“P”) and RNA was subjected to RT-PCR analysis using intronic primers. Readings were normalized to intronic β-actin RNA. (C) Map showing WT PGC-1α and the L2/L3 mutant with the positions of the LXXL motifs indicated (top), and western blot analysis of WT PGC-1α and the L2/L3 mutant expression in LX2 cells (bottom). (D) RT-PCR analysis of cell adhesion gene expression in LX2 cells infected with either an empty vector control adenovirus (“V”), a PGC-1α encoding adenovirus (“P”), or an adenovirus encoding the L2/L3 mutant of PGC-1α (“L2/L3”). Readings were normalized to β-Actin mRNA.

The results described thus far suggest a role for PGC-1α in the regulation of cell adhesion genes in diverse cell types. However, it was unclear whether the observed effects reflect regulation at the level of transcriptional or, given a previous report of an effect of PGC-1α on mRNA processing [21], at the level of mRNA stability. To examine possible mechanisms underlying the effect of PGC-1α on cell adhesion gene mRNAs, we first measured the mRNA stability of cell adhesion gene mRNAs in the presence of PGC-1α expression in LX2 cells. Thus, by using actinomycin D treatment to block transcription in these cells, we were able to determine the half-lives of relevant mRNAs. Interestingly, PGC-1α expression in these cells did not result in a decrease in cell adhesion gene mRNAs, and in fact somewhat increased the stability of several relevant mRNAs (Fig 5A). Therefore, effects on mRNA stability cannot explain the observed down-regulation of cell adhesion gene expression in the presence of PGC-1α.

As intronic RNA expression is believed to reflect transcription rates in cells [22, 23], we examined the expression of cell adhesion gene intronic RNA in LX2 cells in the presence of PGC-1α. Importantly, PGC-1α elicited down-regulation of cell adhesion gene intronic RNA expression (Fig 5B), consistent with an effect on transcription of these genes.

Since PGC-1α is known to exert many of its transcriptional effects through its nuclear hormone receptor binding LXXL motifs, we generated an adenovirus containing PGC-1α with mutated L2 and L3 LXXL motifs (“L2/L3”, Fig 5C, top) in an attempt to examine a possible involvement of these motifs in the PGC-1α mediated down-regulation of cell adhesion genes. Expression of the L2/L3 mutant in LX2 cells yielded levels of expression similar to those observed with WT PGC-1α (Fig 5C, bottom). As expected, the L2/L3 mutant was deficient in its ability to boost the expression of a known target for PGC-1α gene activation ability, COX7B (Fig 5D). Interestingly, the L2/L3 motif was partly required for inhibition of several cell adhesion genes by PGC-1α, as it was still able to exert some inhibitory effect on several cell adhesion mRNAs (Fig 5D).

**Fig 6:**
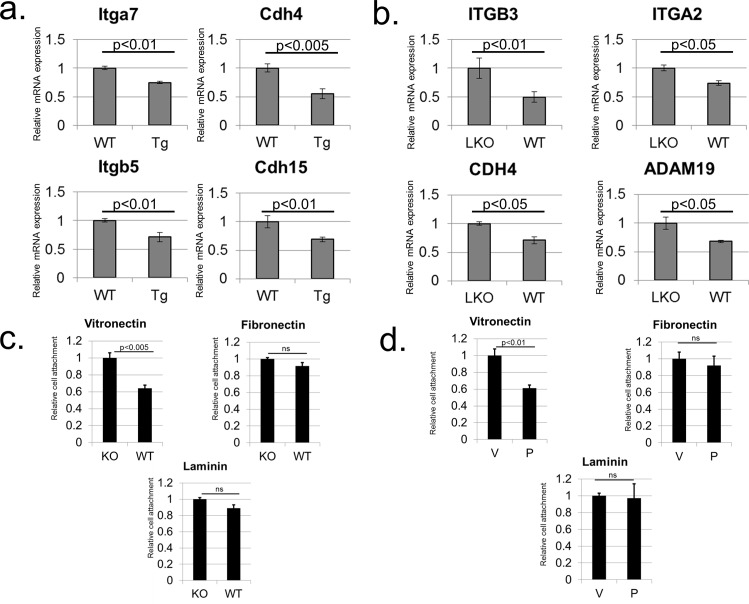
PGC-1α modulates cell adhesion to extracellular matrix components and cell adhesion gene expression *in vivo*. (A) Cell adhesion gene expression in gastrocnemius muscle of WT (“WT”) mice and mice expressing a PGC-1α transgene under a muscle-specific promoter (“Tg”). Values represent averages of data from 5–6 mice. (B) RT-PCR analysis of mRNA expression of several cell adhesion genes in mouse liver during fasting. Values represent averages of data from 8–9 mice. (C) KO or WT PGC-1α MEFs were dissociated and plated in wells pre-coated with the indicated extracellular matrix molecules. Attached cells were stained with crystal violet and scored. n = 7 for all measurements. (D) LX2 cells were infected with either an empty vector control adenovirus (“V”) or a PGC-1α encoding adenovirus (“P”). Cells were dissociated and plated in wells pre-coated with the indicated extracellular matrix molecules. Attached cells were stained with crystal violet and scored. n = 7 for all measurements.

To further establish PGC-1α effect on cell adhesion *in vivo*, as well as to examine PGC-1α effects under more physiological conditions relevant for PGC-1α metabolic functions, we used two relevant mouse models. First, we utilized a mouse with a muscle specific PGC-1α ectopic expression, widely used as a model for exercise-related PGC-1α effects [6, 20]. Comparing WT and transgenic (Tg) mice revealed a highly statistically significant PGC-1α dependent inhibition of expression of several cell adhesion related genes (Fig 6A). Furthermore, as PGC-1α is known to be induced during fasting in liver, we used liver specific PGC-1α knockout (LKO) mice and demonstrated that under fasting conditions PGC-1α can down-regulate the expression of several cell adhesion genes in liver as well (Fig 6B).

Since our study revealed an effect of PGC-1α on expression of cell adhesion genes, we explored the possibility of a corresponding effect of PGC-1α on cell adhesion (e.g., adhesion to extracellular matrix proteins). For that purpose, we set up cell adhesion assays and examined the ability of cells to adhere to surfaces coated with various extracellular matrix components. Intriguingly, we noticed a highly statistically significant reduction in the ability of PGC-1α KO MEFs to attach to vitronectin (Fig 6C). Notably, a similar reduction in the ability of PGC-1α KO MEFs to adhere to other extracellular matrix components such as fibronectin and laminin was not observed (Fig 6C). In addition, LX2 cells expressing PGC-1α bound less avidly to a Vitronectin coated surface (Fig 6D). These observations provide a proof of principle that PGC-1α can affect cell adhesion.

Thus, PGC-1α can elicit down-regulation of cell adhesion gene expression at the transcriptional level, and modulate cell attachment to extracellular matrix components.

## Discussion

PGC-1α acts as a key metabolic regulator, controlled by environmental signals such as cold, scarce food (fasting) and exercise to activate relevant metabolic transcriptional networks, allowing for proper cellular and physiological response. Interestingly, recent studies raise the possibility that it can also act to inhibit gene expression in other scenarios [16, 17].

The work described herein uncovers a surprising connection between PGC-1α, a pivotal metabolic co-activator, and a transcriptional program controlling cell adhesion gene expression. Our results broaden the scope of known targets for PGC-1α activity and further implicate PGC-1α in down-regulation of gene expression. The effects of PGC-1α are exerted on a limited number of cell adhesion molecules (e.g. Fig 1A and Fig 2A), suggesting it is a specific, rather than a global regulator of cell adhesion gene expression.

It is noteworthy that several of the cellular systems used in this study consist of fibroblastic cells, a relatively unexplored cell type for PGC-1α activity. With regard to MEFs, there is some evidence that these cells depend on PGC-1α for activation of anti-oxidative stress related genes under certain conditions [24]. Our findings reveal that endogenous PGC-1α down-regulates numerous cell adhesion related genes in these cells under basal conditions, allowing prospective studies to focus on fibroblasts as a possible cellular context for PGC-1α activity. While we observe prominent effects of endogenous PGC-1α status on cell adhesion gene expression in both MEFs and brown adipocytes, it is conceivable that there is some redundancy in that regard with its family member, PGC-1β. Such redundancy may mask the full effect of PGC-1 family members on cell adhesion and will be the subject of future research. In addition, while some cell adhesion targets were consistently down-regulated by PGC-1α among all or most cell types tested (such as cdh11, pcdh18 or adam23) several others were down-regulated in a few or one cell type, potentially reflecting cell type-specific regulation.

PGC-1α null mice have several developmental phenotypes, including stunted growth [25] and defective perinatal maturation of the heart [26]. Our study points to an effect of PGC-1α on cell adhesion gene expression in multiple cell types. The exact significance of these novel findings *in vivo* remains to be established. Possibly, PGC-1α regulation of cell adhesion gene expressionaffects some of the developmental phenotypes described above. Alternatively, PGC-1α may affect specific cell adhesion related processes beyond development, such as fibrosis following injury, where fibroblasts play key roles. These effects may be regulated via known metabolic cues that modulate PGC-1α levels and activity, allowing for fine-tuning cell adhesion in response to metabolic signals. Alternatively, even in the absence of specific metabolic cues, basal levels of PGC-1α might be sufficient to elicit an effect on cell adhesion gene expression.

What could be the mechanisms at play in mediating the effects of PGC-1α on cell adhesion gene regulation? Previous studies suggested PGC-1α may be involved in regulation of both transcription and mRNA processing [21]. Conversely, our data is consistent with a transcriptional effect on cell adhesion gene expression as PGC-1α expression elicits changes in intronic RNA expression (Fig 5B). Furthermore, PGC-1α expression was unable to induce destabilization of several cell adhesion mRNAs (Fig 5A).

PGC-1α may exert its transcriptional effects directly or indirectly. Recent studies indicate that at least some of the observed anti-inflammatory activity of PGC-1α may be indirectly affecting the transcriptional activity of NF-κB [16]. It thus is possible that some of the effects observed in our study are mediated indirectly through NF-κB or other factors. However, our MEF microarray analysis did not reveal a clear effect of endogenous PGC-1α on metabolic gene expression in these cells under the conditions used. This observation raises the possibility that potent co-activation of metabolic genes by PGC-1α in this system is not the indirect driver of the observed inhibition of cell adhesion gene expression. Alternatively, it is conceivable that PGC-1α directly inhibits transcription of cell adhesion genes, likely through an interaction with a transcription factor, as PGC-1α is not known to possess a direct DNA-binding capacity. Indeed, our recent work revealed that PGC-1α can directly suppress the activity of HSF1 [17]. While so far we are unable to support a role for HSF1 in the observed effects of PGC-1α on cell adhesion gene expression, HSF1 or additional DNA-bound factors may mediate the inhibitory effects of PGC-1α. In this regard, it is interesting to note that the L2/L3 motifs of PGC-1α were only partly required to mediate the down-regulation of cell adhesion genes (Fig 5D). This finding points to the possibility that additional DNA-bound factors beyond nuclear hormone receptors mediate some of the observed effect of PGC-1α on cell adhesion gene expression. In addition, the L2/L3 mutation could affect the structure of the PGC-1α protein and thus modulate the interaction of PGC-1α with partners other than nuclear hormone receptors, impacting the regulation of cell adhesion gene expression.

PGC-1α regulates adhesion to vitronectin (Fig 6C and 6D). It is conceivable that the concerted action of several cell adhesion gene targets regulated by PGC-1α underlies this effect. Alternatively, regulation of a specific target gene by PGC-1α (e.g. an integrin) can be critical to mediating PGC-1α effects on adhesion to a specific ECM adhesion molecule such as vitronectin. Future studies with knockouts/knockdowns of specific candidate genes can determine whether regulation a single target gene is sufficient for the observed effects of PGC-1α on cell adhesion.

In conclusion, our work uncovers a link between a central pathway of metabolic regulation and cell adhesion gene regulation, pointing to a yet unappreciated aspect of PGC-1α activity. Future studies will focus on uncovering possible mechanisms and extending these observations to other systems.

## Materials and Methods

### Cell culture and treatments

LX2 cells were a kind gift from Dr. Scott Friedman, Mount Sinai Hospital, and grown in EMEM containing 10% FBS and antibiotics. Immortalized brown preadipocytes were a kind gift from Dr. Bruce Spiegelman, Harvard Medical School, and were grown in DMEM containing 10% FBS and antibiotics. Immortalized brown preadipocytes were differentiated as described [19]. C2C12 cells (ATCC CRL-1772) were grown in DMEM containing 20% FBS and antibiotics, and differentiated into myotubes in DMEM supplemented with 2% donor equine serum and 1μM insulin for 7 days. Primary MEFs were a kind gift from Dr. Daniel Kelly, Medical College of Wisconsin, and were maintained in DMEM containing 10% FBS and antibiotics. Primary human hepatic stellate cells were from a 54 year old female donor and were obtained from ZenBio. Adenoviruses were generated and produced in HEK293 cells as previously described [27] and added directly to the culture medium where indicated. Actinomycin D was from Sigma (A1410) and was used at 2μg/ml in culture medium.

### Gene expression analysis

RNA was extracted using the RNeasy kit from Qiagen. RNA was reverse-transcribed using the Superscript III First Strand Synthesis kit from Invitrogen. Real-time PCR was performed on an Applied Biosystems 7300 Real Time PCR System using QuantiTech SYBR Green mix. Complete primer information is available as S1 Table. Microarray analysis was performed using the Affymetrix GeneChip Gene arrays according to manufacturer’s instructions. The data expressed as CEL files were normalized by the robust multiarray average (RMA) method with the Expression Console software (Affymetrix), and deposited on the GEO as GSE81171. Gene annotation enrichment analysis was performed using the Gene Ontology Consortium enrichment analysis tool (http://geneontology.org/page/go-enrichment-analysis) with the Bonferroni correction to control for multiple testing.

### Antibodies

Antibodies used were FLAG (Sigma F3165), β-Actin (Santa-Cruz sc-47778), Integrin α2 (BD 611016) and Integrin β5 (Assay Biotech C0235).

### Mice

All animal experiments were performed according to procedures approved by the IACUC of the Rockefeller University. Mice were on a standard chow diet and housed in a pathogen-free facility under a standard 12 hr-light, 12 hr-dark cycle. Seven to nine weeks old male mice were used for experiments.

### Cell adhesion assays

Cell adhesion assays were preformed using Millicoat coated strips from Millipore according to manufacturer’s instructions. Briefly, cells were dissociated in the presence of TrypLE Express Enzyme (ThermoFisher 12604013) and approximately 2.5×10^4^ cells were plated in a single rehydrated well of a 96-well plate pre-coated with the indicated extracellular matrix components. Cells were incubated for 30 minutes, washed, and stained with Crystal Violet. Following additional washes and extraction, absorbance was measured at 590nm. Measurements from BSA coated wells were used as negative controls and subtracted from experiment well measurements. Poly-L-Lysine coated wells served as positive controls to monitor equal cell loading, and experiment measurements were normalized to measurements from Poly-L-Lysine coated wells.

## Supporting Information

S1 Fig Inhibition of cell adhesion gene expression by PGC-1α in primary human hepatic stellate cells. RT-PCR validations of several cell adhesion genes identified in the primary human hepatic stellate cell microarray analysis as down-regulated dependent on PGC-1α. Cells were infected with either an empty vector control adenovirus (“V”) or a PGC-1α encoding adenovirus (“P”). Readings were normalized to β-Actin mRNA.
(TIF)

S2 Fig Endogenous PGC-1α inhibits cell adhesion gene expression in brown adipocytes. (A) Reanalysis of publicly available microarray data from brown adipocytes cells (GSE5041). Shown are cell adhesion related genes down-regulated 1.3 fold or more dependent on PGC-1αbetween each KO and WT sample. (B) Gene annotation enrichment analysis of genes down-regulated 1.5 fold or more dependent on PGC-1α in the brown preadipocyte microarray reanalysis.
(TIF)

S3 Fig PGC-1α activates mitochondrial gene expression in brown adipocytes. Gene annotation enrichment analysis of genes up-regulated 1.5 fold or more dependent on PGC-1α in PGC-1α KO brown adipocytes infected with an adenovirus encoding PGC-1α.
(TIF)

S1 Table List of primers used in this study for RT-PCR. (XLSX)
