# Supplementary figures and images for: Inhibition of Adhesion Molecule Gene Expression and Cell Adhesion by the Metabolic Regulator PGC-1α
Source: PLoS One. 2016 Dec 16;11(12):e0165598. doi: 10.1371/journal.pone.0165598 (PMC5161318; doi:10.1371/journal.pone.0165598)

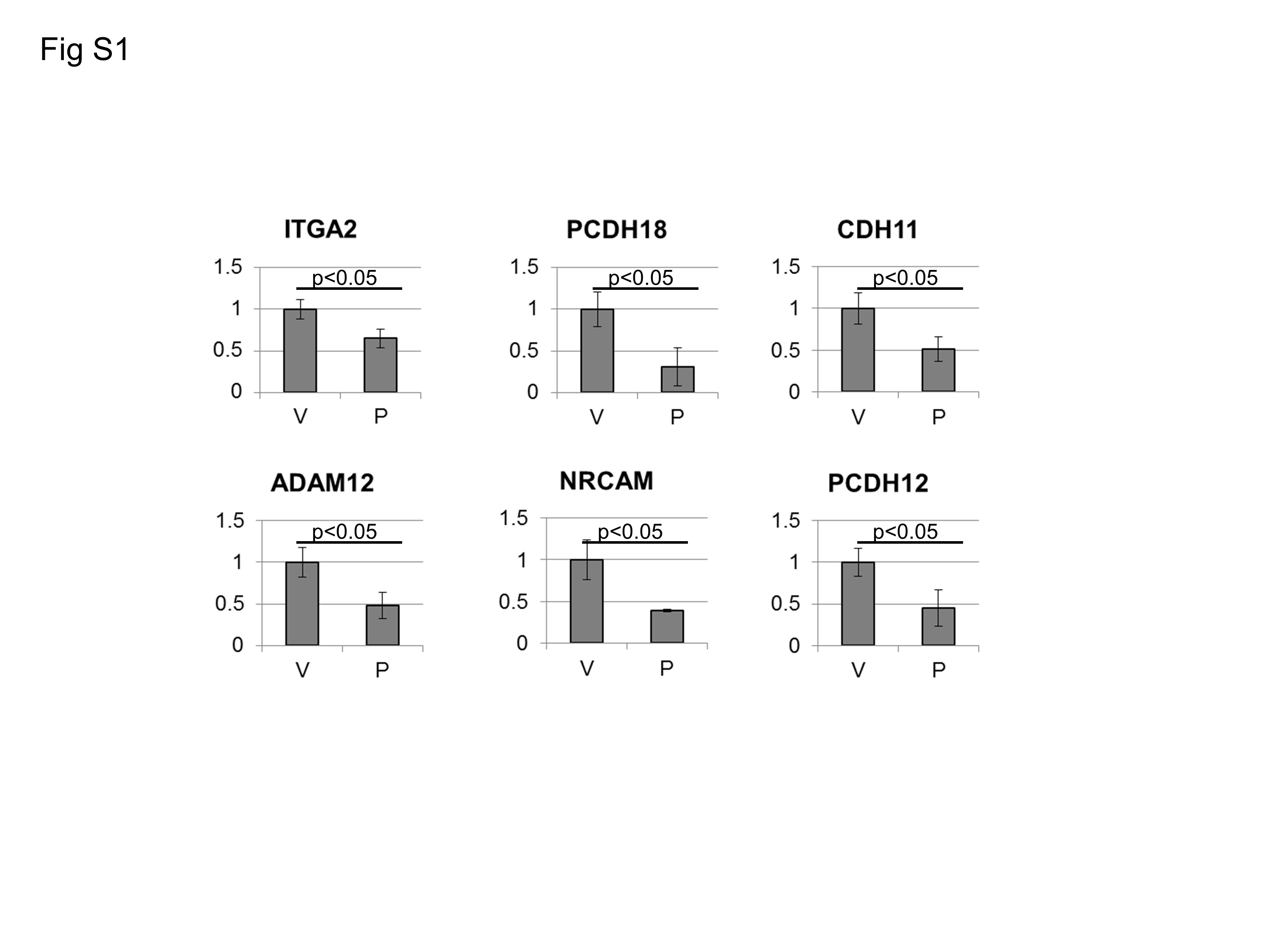

Supplement: S1 Fig — RT-PCR validations of several cell adhesion genes identified in the primary human hepatic stellate cell microarray analysis as down-regulated dependent on PGC-1α. Cells were infected with either an empty vector control adenovirus (“V”) or a PGC-1α encoding adenovirus (“P”). Readings were normalized to β-Actin mRNA. (TIF) [file pone.0165598.s001.tif]

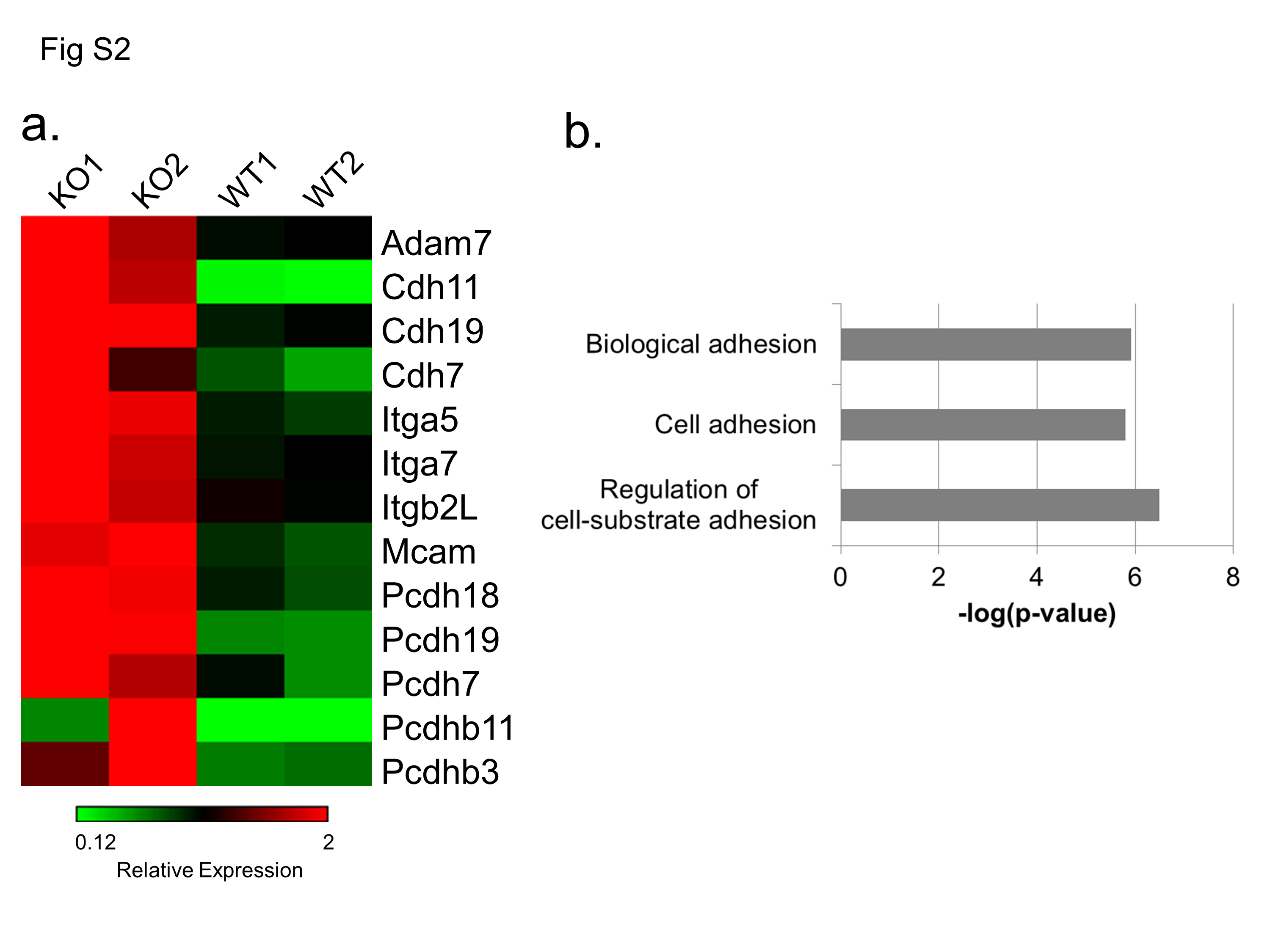

Supplement: S2 Fig — (A) Reanalysis of publicly available microarray data from brown adipocytes cells (GSE5041). Shown are cell adhesion related genes down-regulated 1.3 fold or more dependent on PGC-1αbetween each KO and WT sample. (B) Gene annotation enrichment analysis of genes down-regulated 1.5 fold or more dependent on PGC-1α in the brown preadipocyte microarray reanalysis. (TIF) [file pone.0165598.s002.tif]

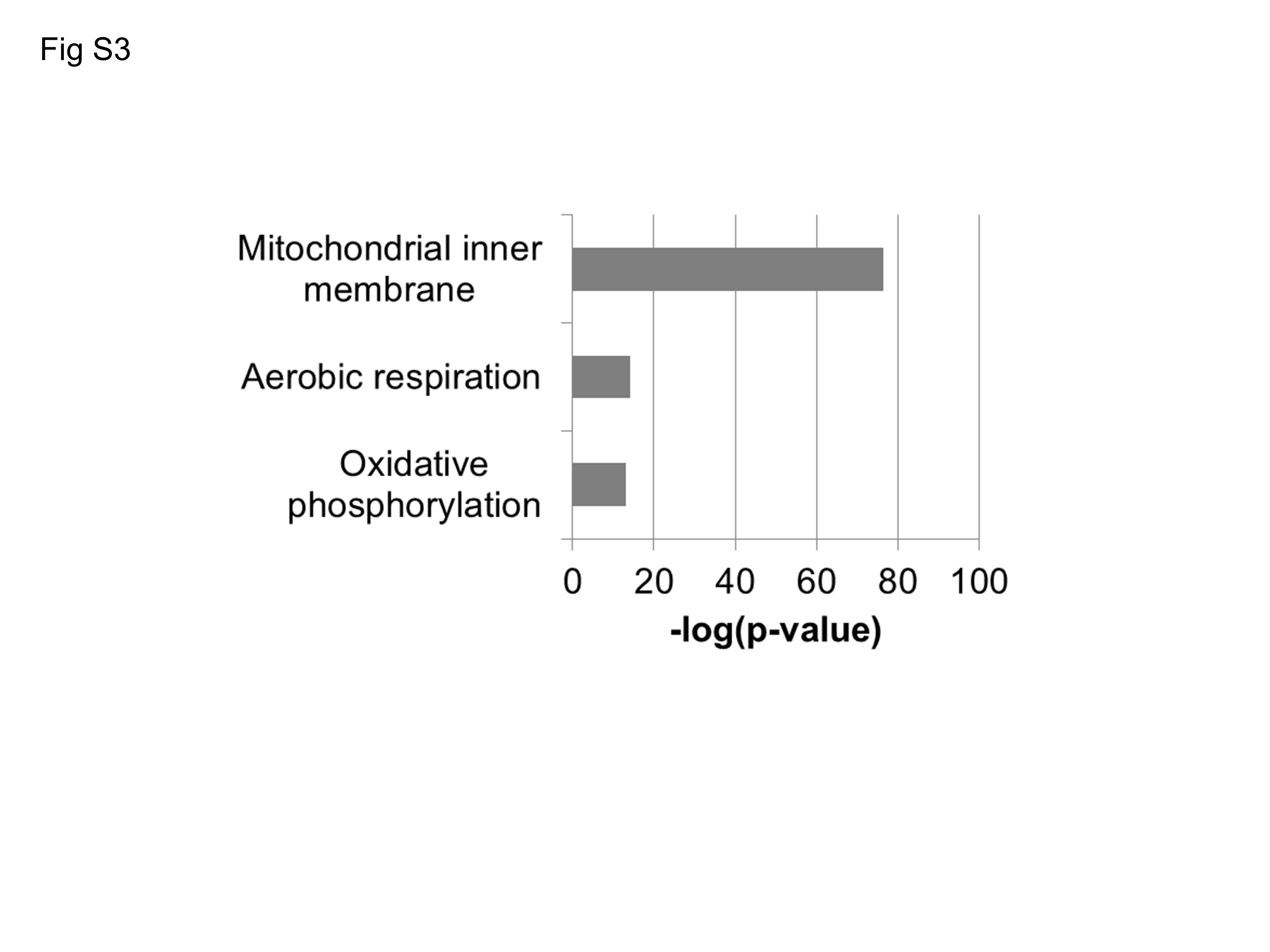

Supplement: S3 Fig — Gene annotation enrichment analysis of genes up-regulated 1.5 fold or more dependent on PGC-1α in PGC-1α KO brown adipocytes infected with an adenovirus encoding PGC-1α. (TIF) [file pone.0165598.s003.tif]
